# Toward Precision Health in Autoimmunity and Immune-Related Adverse Events: The Autoantibody Reactome, Spatial Omics, and Multimodal Data Integration

**DOI:** 10.3390/biomedicines14051129

**Published:** 2026-05-16

**Authors:** Allan Stensballe

**Affiliations:** 1 Department of Health Science and Technology, Faculty of Medicine, Aalborg University, Gistrup, 9260 Aalborg, Denmark; allan@stensballe.com; Tel.: +45-61608786; 2 Clinical Cancer Research Center, Aalborg University Hospital, Gistrup, 9260 Aalborg, Denmark

**Keywords:** autoimmunity, immune-related adverse events, rheumatoid arthritis, immune-checkpoint inhibitors, autoantibody reactome

## Abstract

The autoantibody reactome refers to the multidimensional repertoire of antibody reactivities against self-antigens across the human proteome or selected antigenic compartments. This offers a scalable systemic layer for precision immunology across spontaneous autoimmunity and treatment-induced immune toxicity. Autoimmune diseases and immune-related adverse events (irAEs) share major features of dysregulated immunity, yet clinically useful tools for risk stratification, early detection, endotyping, and treatment guidance remain limited and slow. A central challenge is that tissue pathology is highly informative but not uniformly accessible across diseases and organ systems, whereas routine serology captures only a narrow fraction of immune heterogeneity. In this perspective, I argue that a global autoantibody reactome can serve as a central unifying framework linking systemic immune history, tissue pathology, and clinical trajectories across autoimmune disorders and irAEs. Rheumatoid arthritis (RA) provides a strong prototype because its serological diversity, major role of post-translationally modified autoantigens, and marked synovial heterogeneity allow reactome features to be interpreted against tissue biology. Immune checkpoint inhibitor-associated inflammatory arthritis serves as an illustrative rheumatic irAE and a model of treatment-induced immune dysregulation with clear opportunities for longitudinal blood-based profiling. Spatial transcriptomics and proteomics are therefore positioned not as stand-alone solutions, but as mechanistic tools that can decode reactome-defined immune states within tissue microenvironments where tissue is accessible. Clinical translation will require integration of autoantibody reactomes with tissue, circulating proteomic, imaging, genetic, and clinical data through transparent multimodal models, as well as a shift from exploratory resources such as AAgAtlas toward analytically validated and clinically interpretable biomarker panels for risk prediction, endotyping, monitoring, and biomarker-guided intervention. This perspective outlines technical and strategic steps toward clinically actionable decision support, including risk stratification before ICI initiation and treatment guidance for patients who develop ICI-induced inflammatory arthritis, through integration of autoantibody reactome profiling, spatial omics and transparent multimodal AI.

## 1. Introduction: The Autoantibody Reactome as a Unifying Framework Across Inflammation, Autoimmunity and irAEs

**Figure 1:**
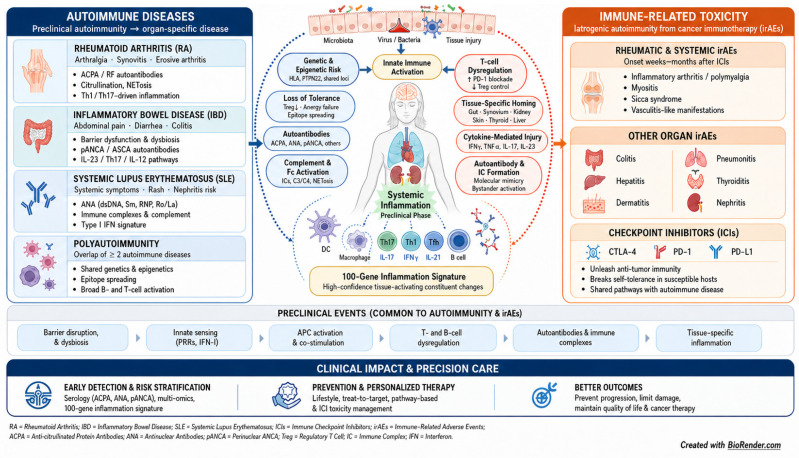
Autoimmune, autoinflammatory and immune-related toxicity-driven inflammation range from preclinical immune events to disease manifestations and precision health biomarker opportunities. A continuum from early inflammatory and immune-perturbing events to clinical manifestations in common autoimmune diseases and immune-related adverse events (irAEs) are linked to immune events, specific immune cell signaling and potential complex hallmark inflammation biomarkers. The core inflammatory signature across inflammatory or autoimmune disorders can be condensed into a 100-gene product signature. Created in BioRender. Stensballe, A. (2026) https://BioRender.com/pb8lhom (accessed on 4 May 2026).

Autoimmune- and immune-mediated inflammatory diseases display marked heterogeneity in onset, trajectory, and treatment response [1,2]. Inflammation is an evolutionarily conserved biological response triggered by harmful stimuli such as pathogen invasion, tissue injury, or cellular damage and associated with a high-confidence subset of 100 gene products capturing the most consistent changes [3]. Humoral immune mechanisms (e.g., complement and antibodies) contribute to inflammatory responses triggered by infection, injury, and cellular damage, but they represent only the B-cell-/antibody-mediated arm of adaptive immunity. While inflammation is vital for clearing harmful stimuli and supporting tissue repair, then persistent or dysregulated inflammation can drive the onset and progression of many conditions, including cardiovascular and metabolic disease [4,5], inflammatory bowel disease (IBD) [6,7], cancer [8], autoimmune disorders [9], and cardiovascular diseases [10]. At the same time, modern cancer immunotherapies, particularly immune checkpoint inhibitors (ICIs), have transformed oncology but introduced a new class of treatment-induced immune toxicities collectively termed immune-related adverse events (irAEs) [11,12,13] (Figure 1).

Autoantigens (AAgs) are endogenous proteins that are targeted by humoral antibodies known as “autoantibodies” (AAbs). The detection of serological AAbs has been used to diagnose autoimmune diseases over the last 40 years going from single biomarkers to complex biomarker patterns [14]. Now, more than 8000 non-redundant autoantigens and clinically relevant post-translationally modified AAgs can be tested for >1050 human diseases [15]. AAbs have long been used as valuable diagnostic and prognostic biomarkers in autoimmune disease as single biomarkers (e.g., RF). However, AAbs are increasingly better understood as part of a broader systems-level immune readout and key variables in complex biomarkers schemes. The autoantibody reactome defines the multidimensional repertoire of antibody reactivities against self-antigens across the human proteome or selected antigenic compartments. It extends beyond single diagnostic autoantibodies to capture the breadth, intensity, specificity, dynamics, and functional consequences of humoral autoreactivity in health-to-disease transitions, autoimmune diseases with overlapping symptoms, cancer, and immune perturbation. The international community extend the global autoantibody reactome as an opportunity for scalable systems-level readout that can connect systemic autoreactivity to specific disease endotypes and tissue pathology across autoimmune disorders and shared AAgs. In this framework, rheumatoid arthritis (RA) serves as a prototype tissue pathology in which systemic reactome signals can be interpreted against synovial biology, whereas irAEs serve as induced models of immune dysregulation that are well suited for longitudinal biomarker studies. Rheumatic irAEs, particularly ICI-induced inflammatory arthritis, are among the most frequent chronic toxicities and can substantially affect quality of life and cancer treatment decisions [12,16,17,18]. Together, these contexts motivate a precision health strategy that integrates autoantibody reactomes with tissue profiling, circulating serology biomarkers, imaging, and clinical metadata.

Rheumatic diseases encompass a wide range of disorders benefitting from complex biomarkers for individualized diagnostics and prognostics, including RA [19,20], systemic lupus erythematosus (SLE) [21,22,23], psoriatic arthritis (PsA) [24,25], and polymyalgia rheumatica (PMR) [26,27,28]. Most rheumatic diseases are characterized by systemic inflammation that drives joint destruction, tissue damage, multi-organ complications and systemic comorbidity [29,30]. Autoimmunity is also central to their pathogenesis, and autoantibodies are important markers for diagnosis and monitoring disease progression [30,31]. In RA, persistent immune-mediated injury can become irreversible over time, making management increasingly challenging [32].

Despite major advances, clinical decision-making still relies heavily on composite clinical scores and simple serology, while biologic and targeted synthetic disease-modifying antirheumatic drugs are typically prescribed in stepwise algorithms that only partially account for molecular heterogeneity [33]. Both genetic and environmental factors contribute to RA disease etiology, with genetic predispositions accounting for up to 50% of the overall disease risk [34]. A key clinical factor differentiating RA patients is the presence of anti-citrullinated protein antibodies (ACPAs), with ACPA-positive patients having a more aggressive disease course and higher relapse rates. Despite this clear clinical stratification, the molecular mechanisms underlying RA heterogeneity in distinct disease phenotypes remain incompletely understood. This “precision gap” reflects the mismatch between complex, spatially organized synovial pathology on the one hand and relatively coarse treatment stratification on the other hand [35].

ICI-induced inflammatory arthritis offers a complementary view of autoimmunity. Here, a defined therapeutic trigger perturbs immune tolerance, and a subset of patients develops arthritis that shares features with, but is not identical to, classical RA [12,36,37,38]. Some patients experience self-limited arthritis that mirrors the course of cancer therapy, whereas others develop chronic, erosive disease requiring long-term rheumatologic care [18]. The factors determining who develops chronic arthritis, and who remains unaffected, are incompletely understood.

Across these contexts, there is growing recognition that (1) complex biomarker profiles (e.g., miRNA; mRNA; proteins; cytokines; extracellular vesicles (EVs)) support precision health-based profiling [39,40,41], (2) spatially resolved tissue omics can reveal pathogenic microenvironments and dynamic tissue states associated with disease course and treatment response [42], (3) autoantibody reactomes provide high-dimensional, systemic fingerprints of immune history and predisposition to autoimmunity and toxicity [31,43,44,45,46], and (4) AI-enabled multi-OMICs integration and inflammatome profiling are required to fuse these complex layers into actionable precision health tools [3,45,47,48,49]. The following sections discuss how RA can function as a pathology-anchored prototype, how autoantibody reactomes provide a scalable systemic readout, and how multimodal integration may translate these layers into clinically useful tools across autoimmunity and irAEs.

## 2. Rheumatoid Arthritis as Prototype Tissue Pathology for Adverse Effects and Immune-Checkpoint Inhibitor-Induced Arthritis

RA is introduced first because it offers one of the clearest settings in which systemic autoreactivity can be interpreted against accessible, spatially organized tissue pathology. A simplified framework for mapping biomarker layers onto clinical use cases in RA and associated ICI-IA is summarized in Table 1.

### 2.1. Spatial Omics in Rheumatoid Arthritis as a Precision Health Prototype

The RA synovium is a mosaic of interacting fibroblast, myeloid, lymphoid and endothelial cell populations embedded in an evolving extracellular matrix (ECM) [59,64]. Spatial transcriptomics and proteomics technologies, e.g., laser microdissection, microfluidics followed by NGS or LC-MS workflows, now allow gene and protein expression to be measured while preserving the anatomical context of these cells [42,81,82]. In contrast to bulk tissue or blood assays, spatial OMICs capture which cells reside next to each other, which pathways are active in specific microenvironments and how this architecture changes with disease stage and therapy [75].

Recent studies have generated high-resolution atlases of RA synovium, identifying fibroblast populations enriched at the pannus–bone interface, distinct myeloid niches, and lymphoid aggregates consistent with tertiary lymphoid structures. These findings show that RA tissue pathology is not uniform but organized into recurrent cellular neighborhoods with distinct biological functions [59,60,61]. TLSs mainly consist of highly organized clusters of lymphocytes not formed in secondary lymphoid organs (SLOs) that can vary with the nature of the autoimmune disorder. Imaging mass cytometry (IMC) and related high-dimensional approaches further support the view that RA synovium contains spatially structured immune–stromal programs that may help explain differences in disease progression and treatment response [62]. Looking ahead, deep learning approaches applied to spatial and single-cell datasets may help define reproducible synovial states that extend beyond conventional histological classification.

From a precision health perspective, spatial omics in RA has three main applications. First, in at-risk individuals, early synovial changes may help identify preclinical states before overt arthritis develops. Second, in established disease, tissue-defined endotypes may clarify why patients differ in response to biologics targeting TNF, IL-6, B cells, or other pathways. Third, in treated disease, serial tissue sampling may reveal whether remodeling of specific pathogenic niches predicts remission, flare, or successful tapering [33].

These opportunities are balanced by practical challenges. Spatial OMICs remain costly and technically intensive, and synovial biopsy is still not standard-of-care in many rheumatology settings. Current datasets are often small and single-center, raising concerns about generalizability and standardization. Nevertheless, RA provides a compelling prototype of tissue-resolved immune landscapes that can close part of the precision gap between molecular pathology and clinical management.

### 2.2. Extending Tissue Profiling to Immune-Checkpoint Inhibitor-Induced Arthritis

Immune-checkpoint inhibition (ICI) offers a distinct but related window on autoimmunity. ICI-induced inflammatory arthritis (ICI-IA) arises in the context of deliberate immune activation against cancer and shows both overlap and divergence with RA [38,63]. ICIs have transformed the therapeutic landscape of oncology but are frequently accompanied by irAEs. ICI-IA has emerged as the most common musculoskeletal toxicity, often mimicking RA in its clinical and imaging features. Histopathological and synovial tissue analyses suggest that T-cell-dominated infiltrates, often enriched for cytotoxic and Th1/Th17-skewed profiles, underlie persistent arthritis in a subset of patients.

Current biomarker studies on ICI-induced inflammatory arthritis have focused largely on circulating cytokines and conventional autoantibodies, with mixed and often non-replicated results [12,13]. Recent studies in cancer immunotherapy suggest that irAEs should be interpreted within a broader immune context, where anti-tumor immunity, autoreactivity, microbial signals and tumor-intrinsic pathways intersect. In urothelial carcinoma, antibiotic exposure has been associated with attenuated responses to immune checkpoint blockade, with mechanistic analyses implicating intratumoral bacteria and CD74–MIF/COPA signaling in the crosstalk between anti-bacterial and anti-tumor immunity [83]. Similarly, FGFR alterations have been linked to reduced ICI responsiveness and an immune-modulated tumor microenvironment, supporting the concept that oncogenic programs can shape the balance between immune activation, immune escape and treatment toxicity [84,85]. Although these studies are not specific to ICI-induced arthritis, they reinforce the need to move beyond isolated blood biomarkers and examine how systemic immune activation is translated into tissue-specific inflammation. Spatial synovial profiling, informed by lessons from RA, could help determine how checkpoint blockade reshapes joint microenvironments and whether pretreatment immune states are linked to later chronic arthritis. When combined with high-dimensional autoantibody profiling, this approach may reveal pre-existing or treatment-emergent autoreactivity patterns that anticipate toxicity before symptoms become clinically apparent. Together, these advances support an integrated framework in which circulating immune biomarkers, tumor–microbiome interactions and spatial tissue pathology are studied jointly to define mechanisms of ICI-induced inflammatory arthritis.

Therapeutic strategies balance effective arthritis control with preservation of anti-tumor immunity [86]. Glucocorticoids and conventional disease-modifying anti-rheumatic drugs (cDMARDs) are mainstays with established safety and efficacy, while biologic therapies are typically reserved for severe or refractory disease, largely due to concerns about potential effects on cancer progression [50,63]. Patients receiving ICIs often have structured sampling schedules, including baseline and on-treatment blood draws and systematic imaging. Integrating synovial spatial omics, imaging and machine learning with electronic health records (EHRs) into such cohorts would enable genuinely longitudinal, multi-layered studies of inflammatory arthritis, with clear timing of the immune insult and defined clinical endpoints [51,87]. This makes ICI-induced arthritis an ideal test bed for tissue-informed precision rheumatology.

## 3. Autoantibody Reactomes as Scalable Systemic Immune Readouts

Autoantibodies are traditionally viewed as pathogenic or diagnostic markers in autoimmune disease, but they are increasingly appreciated as part of a broader immunological sensing system [52,53]. Such signatures can precede clinical autoimmunity by years, as shown in diseases such as systemic lupus erythematosus and RA, and can provide early warning of disease, molecular subtype and prognosis [30,31,88]. Arthralgia, an early manifestation preceding definite RA, represents a critical window to identify high-risk individuals and implement timely interventions [89]. The transition from arthralgia to established RA remains incompletely defined and the underlying immunopathological mechanisms will be important to establish as part of the reactome enabling early preclinical assessment.

Profiling autoantibodies in patient samples at scale depends on standardized proteomic technologies and curated autoantigen resources [54,90]. High-density protein microarrays, including those incorporating native or post-translationally modified proteins, can assess reactivity against thousands of potential autoantigens in a single experiment [45,91]. In contrast to many other autoimmune conditions, RA-associated autoantibodies recognize both unmodified and a range of post-translationally modified protein epitopes, collectively termed anti-modified protein antibodies (AMPAs). Among these, ACPAs are the most specific and exhibit >90% specificity for RA. Other AMPAs, including anti-carbamylated protein antibodies (anti-CarP), anti-acetylated protein antibodies (AAPA), and anti-malondialdehyde–acetaldehyde adduct antibodies (anti-MAA), are less specific than ACPAs but are linked to inflammatory activity and may contribute to disease progression [57]. AAgAtlas and related tools can provide comprehensive, literature-curated catalogs of human autoantigens that are invaluable for interpreting such profiling data and for prioritizing candidates for diagnostic development [88]. A citrulline-specific protein array containing over one thousand native human proteins citrullinated directly on the array has been used to map anti-citrullinated protein antibody repertoires in RA, distinguishing anti-CCP-positive and anti-CCP-negative patients and revealing hundreds of differentially targeted autoantigens [30,31]. On-array citrullination protocols further demonstrate how neo-epitopes can be generated in situ to mimic in vivo antigen modification, enabling high-throughput discovery of disease-relevant targets [31,58]. The broader concept of protein array-based companion diagnostics then bridges from discovery platforms to focused diagnostic panels that can be deployed in clinical laboratories and in vitro diagnostics-enabling panels [45].

For precision health, the autoantibody reactome offers several opportunities [53,54,55]. First, in individuals at risk for autoimmunity, broad autoantibody panels may improve risk stratification beyond conventional markers such as rheumatoid factor and anti-citrullinated protein antibodies. Second, in established disease, autoantibody profiles can define molecular endotypes that correlate with specific pathways, comorbidities or treatment responses. Third, in the context of ICIs and other immunotherapies, baseline and early on-treatment autoantibody reactomes may identify patients at increased risk of specific irAEs, including inflammatory arthritis, and might also correlate with anti-tumor efficacy. Because autoantibody profiling is minimally invasive and scalable, it is an attractive cornerstone for precision health tools that span both rheumatology and oncology [56].

At present, multiple limitations must be addressed. Initially, exploratory studies are conducted on large cohorts [54,56,89,92] as these are necessary studies, but they cannot currently be adapted for personalized care of individual patients. Expert-center workflows with synchronized biospecimen collection, assay timing, data processing and interpretation will be required to reduce temporal mismatch and support disease-specific validation. Autoantibodies of unclear clinical significance, identified in a specific patient, must be avoided in the context of personalized care for that patient. Their relevance must first be established through large-cohort studies. The intended near-term clinical scenario is therefore not routine spatial–omics profiling of every patient, but risk stratification and mechanistic endotyping in selected high-risk RA/ICI cohorts, with eventual translation into focused companion diagnostic panels. Thus, the transition to an integrative system for clinical decision support at the present time requires validation and multimodal data integration. A further limitation is that the autoantibody reactome primarily captures the humoral/B-cell arm of adaptive immunity; therefore, it should not be interpreted as a complete immune state, but integrated with T-cell, myeloid, stromal, tissue–pathology and clinical data.

## 4. Multimodal Integration: Tissue, Blood, Imaging, and Clinical Data

No single data layer can explain why one individual develops RA, another develops ICI-induced arthritis, and another remains clinically unaffected under comparable exposures. Real-life data layers may be generated by multiple autoantibody profiling technologies enabling broader mapping of the human autoantibody reactome. High-throughput platforms such as PhIP-seq support scalable discovery of autoreactivity across large peptide libraries, while complementary approaches including protein/peptide arrays, REAP and MIPSA capture overlapping but distinct parts of the antibody repertoire, including extracellular, secreted and conformationally preserved antigens [91,93]. Paired single-cell RNA and B-cell receptor sequencing now link B-cell phenotypes, clonal expansion and antigen specificity at cellular resolution [94]. In RA and ICI therapy, these approaches help define patient-specific autoreactive repertoires and suggest that humoral immune features, including functional autoantibodies and B-cell states, may contribute to both treatment response and immune-related adverse events. Spatial and single-cell multi-OMICs further show that irAEs are shaped by local tissue immune niches. In checkpoint inhibitor colitis, single-cell and spatial studies have linked epithelial barrier dysfunction, checkpoint-bound T cells and tissue-resident memory T-cell programs to damaged epithelial microdomains [65,66,67]. Together, these methods support an integrated autoantibody-reactome-framework-based data pool connecting systemic autoreactivity, clonal immune activation and organ-specific pathology.

**Figure 2:**
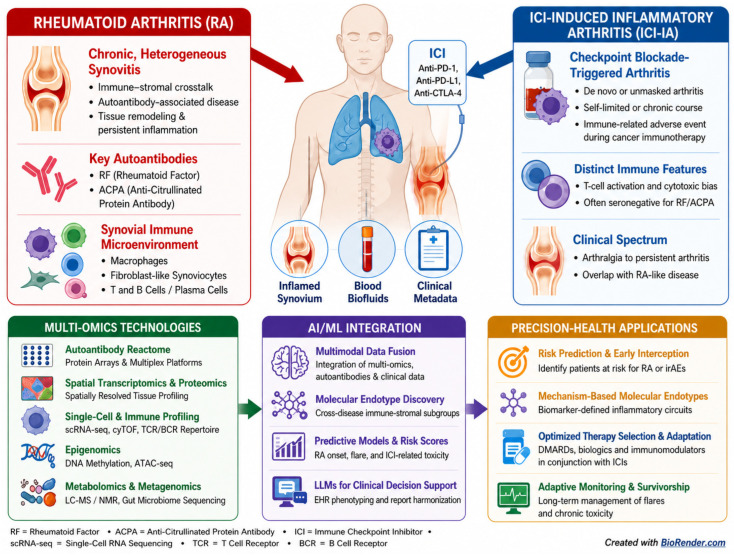
Multi-OMICs and autoantibody integration for precision health in rheumatoid arthritis and ICI-induced inflammatory arthritis. This schematic overview illustrates how rheumatoid arthritis (RA) and immune-checkpoint inhibitor-induced inflammatory arthritis (ICI-IA) can be studied using integrated spatial tissue omics, single-cell immune profiling, autoantibody and circulating proteomic analyses, epigenomics, metabolomics, metagenomics, and clinical/imaging data. These complementary data layers support biomarker discovery, molecular endotyping, risk prediction, therapy selection, and adaptive long-term monitoring. The resulting tools include AI and LLMs support precision health applications, including biomarker-guided clinical decision support, individualized monitoring by smart technology, surveillance strategies and endotype-stratified clinical trial design in both autoimmunity and immune-related adverse events. Created in BioRender. Stensballe, A. (2026) https://BioRender.com/8mc8jlh (accessed on 4 May 2026).

For this reason, precision immunology in autoimmunity and irAEs will depend on multimodal integration rather than on any single biomarker class. The goal is to combine tissue-resolved information, circulating autoantibody and proteomic signals, imaging, genetics, and clinical data into unified models of risk, mechanism, and disease trajectory [41,69]. Multi-OMICs integration seeks to combine (1) spatial transcriptomics and proteomics, (2) circulating proteomic and autoantibody signatures and (3) clinical, imaging and genetic information into unified models of disease risk and progression (Figure 2). Mechanistically, reactome–spatial integration should be interpreted as a staged inference rather than as direct proof of pathogenicity. First, circulating autoantibody profiles identify candidate antigenic targets or autoreactivity patterns. Second, spatial transcriptomic or proteomic data determine whether these targets, their modified forms, or their interacting pathways are enriched in disease-relevant tissue niches. Third, colocalization with inflammatory cell states, stromal programs, complement activation, or tissue damage markers can prioritize antigen–cell–pathway axes for validation. In this way, spatial omics does not simply “confirm” serology, but helps assign systemic autoreactivity to specific tissue compartments and candidate mechanisms.

A broad computational toolkit is available for this purpose, including correlation-based approaches, matrix factorization, network models, Bayesian frameworks, and deep learning architectures such as variational autoencoders and graph neural networks. The value of these methods is not simply technical. Their importance lies in the ability to identify latent disease endotypes, predict future outcomes, and connect systemic autoreactivity to cell-type-specific pathology [70]. In autoimmune diseases, these frameworks are beginning to link genetic risk loci with cell-type-specific expression changes, serum protein signatures and clinical outcomes, thereby revealing coordinated modules of dysregulated immunity. Biomimetic digital twin concepts may further support this framework by integrating multi-OMIC and clinical data into patient-specific models that capture complex disease relationships not apparent from single-modality analyses [71]. Here, complex relationships can be determined that are not readily visible in bioinformatics platforms.

Artificial intelligence (AI) is essential to manage the scale and complexity of these data. In RA, AI methods have been used to classify synovial histology, integrate ultrasound and MRI with clinical data and derive multi-biomarker disease activity scores from proteomic panels. In the ICI setting, machine learning models are being developed that combine baseline clinical characteristics, cytokines, T-cell phenotypes and autoantibodies to predict irAEs and survival.

AI applications in autoimmunity related to RA and irAEs should specify both the model type and dataset used [72,73]. For RA imaging, CNN models such as AlexNet, VGG16 and GoogLeNet have been applied to hand radiographs for diagnosis and staging, while Vision Transformer (ViT)-based models have been used for automated radiographic Sharp/van der Heijde scoring [95,96]. For autoimmune and irAE research, graph neural networks, including graph convolutional networks and graph attention networks, are particularly relevant for modeling immune cell interactions, drug–target–disease networks and shared inflammatory pathways [68]. These AI methods should be linked to disease-relevant datasets, including AMP-RA/AMP RA-SLE resources, which provide synovial scRNA-seq, bulk RNA-seq, mass cytometry, flow cytometry, histology and surface-protein data, as well as emerging single-cell and clinical datasets from immune checkpoint inhibitor-associated irAEs [35,97].

AI-driven integration can be used in at least three ways [74,76,98]. One is endotype discovery, where unsupervised models embed spatial tissue features, cellular composition and autoantibody signatures into a shared latent space to identify endotypes that cut across conventional diagnostic categories, for example RA-like versus non-RA-like arthritis within ICI-induced arthritis. Second is predictive modeling, where supervised models learn relationships between baseline multi-OMICs and future outcomes such as flare risk, radiographic progression or irAE development, providing individualized risk scores that inform preventive or pre-emptive therapy. A third is mechanistic inference, where integrative networks connect autoantibodies, their targets and cell-type-specific expression in tissues, highlighting antigen–cell pairs that may drive pathology in RA or ICI-induced arthritis.

To be clinically useful, such models must be transparent, robust and validated across diverse populations. Interpretability techniques, calibration analyses and external validation cohorts will therefore be essential components of any precision health pipeline in autoimmunity and immune-related toxicity.

## 5. Translational, Infrastructural, and Regulatory Barriers

Biomimetic digital twin concepts may further support this framework by integrating multi-OMIC and clinical data into patient-specific models that capture complex disease relationships not apparent from single-modality analyses [77]. Several barriers must be addressed before autoantibody reactomes, spatial omics, and multimodal models can be translated into clinically useful tools.

Sample acquisition and standardization are major bottlenecks. In RA, synovial biopsy remains technically demanding, particularly in small joints and early disease, and tissue quality may vary substantially across centers. Bringing spatial omics into clinical workflows will therefore require standardized biopsy procedures, harmonized processing pipelines, shared quality metrics, and robust pre-analytical control. Similar standardization challenges apply to high-dimensional autoantibody profiling, where longitudinal stability, batch effects, and assay drift must be carefully managed. Automated ultrasound acquisition and AI-assisted scoring may help standardize imaging endpoints and improve comparability across rheumatology and oncology–rheumatology cohorts [78,79]. At present, a combination of fully automated ultrasound acquisition and AI-based scoring represents a strong step toward standardized, operator-independent ultrasound assessment [74]. One innovative IVD-based approach on personalized autoantibody profiling efforts depends on high-quality, longitudinal sampling with strict pre-analytical control [80].

Endpoint definition and phenotyping must be harmonized. In ICI-induced arthritis, current grading systems and diagnostic categories vary among oncology and rheumatology societies. Inconsistent definitions limit the comparability of cohorts and complicate the training of predictive models. There is a need for consensus criteria that integrate clinical, imaging and, where possible, tissue information and that distinguish self-limited from chronic arthritis.

Data scale and interoperability pose additional technical challenges. Spatial OMICs datasets are large and complex, and autoantibody reactome profiling can generate high-dimensional matrices across thousands of antigens. When combined with electronic health record data and imaging, these resources require substantial computational infrastructure and standardized data models. Interoperable formats and common ontologies are necessary to allow multi-center integration and meta-analysis.

Regulatory and ethical considerations will be equally important. Predictive models for autoimmune disease and irAE risk raise questions about informed consent, communication of probabilistic risk, incidental findings, and the possible impact of biomarker-guided decisions on access to therapy. Any companion diagnostic strategy in this space will therefore require evidence of analytical validity, clinical validity, clinical utility, interpretability, and appropriate post-deployment surveillance.

Concerns about data privacy and security must be addressed to ensure proteomic data are used responsibly. Managing patient-derived proteomic information requires rigorous safeguards to protect confidentiality and ensure compliance with relevant regulatory standards. If these challenges can be overcome—and the strengths of proteomic technologies fully leveraged—precision medicine for inflammatory conditions could become a practical reality. As a leading pillar of multi-OMICs research, proteomics offers the potential for a deeper, more integrated understanding of disease mechanisms, enabling a transformative shift in how inflammatory- and autoimmune-related diseases are treated.

## 6. Toward Clinical Translation

The convergence of spatial omics, autoantibody reactome profiling, and multimodal AI creates a plausible route toward clinically useful precision health tools in autoimmunity and immune-related toxicity. In RA, integrated tissue and blood profiling may refine disease endotypes, support earlier interception, and improve treatment selection. In ICI-induced inflammatory arthritis and other irAEs, longitudinal analyses of circulating biomarkers and, where feasible, tissue pathology, may yield more robust predictors of chronic toxicity, monitoring intensity, and immunomodulatory co-treatment strategies.

Large-scale autoantibody profiling, supported by curated resources such as AAgAtlas and high-content protein array platforms, offers a scalable way to capture long-term immune history and propensity for autoreactivity in a single assay. When these data are connected to tissue-level information from spatial transcriptomics and proteomics, reactome features become biologically interpretable rather than remaining isolated serological measurements.

While autoantibody reactome profiling offers a scalable window into systemic immunity, translation requires careful control of pre-analytical variation, batch effects, and assay drift through rigorous QC and cross-site standards. Reactome features must also demonstrate longitudinal robustness (or predictable dynamics) across treatment changes and intercurrent immune perturbations. Importantly, antigen library design and PTM representation strongly influence measured signatures, motivating standardized PTM chemistry and libraries informed by synovial biology and curated resources such as AAgAtlas. Ultimately, discovery arrays should be distilled into small, analytically validated panels with predefined thresholds and demonstrated incremental clinical utility, enabling focused companion diagnostics for risk stratification, endotyping, and monitoring.

To operationalize an immune “observatory,” AI models should be optimized for clinical deployment rather than retrospective discovery alone, with pre-specified endpoints and outputs that map to decisions. Robustness requires external validation across centers and platforms, alongside calibration so that predicted risks correspond to observed outcomes and can support actionable thresholds. Because dataset shift is expected in real-world settings (assay drift, imaging changes, evolving ICI regimens), models should incorporate drift detection, recalibration strategies, and ongoing post-deployment performance monitoring. A pragmatic approach is to deliver minimal viable outputs such as a risk score, an endotype label, and a monitoring interval, each explicitly linked to a recommended clinical action.

Going forward, key priorities will include (1) designing joint RA–ICI cohorts with standardized biospecimen pipelines, (2) implementing harmonized protein array and autoantibody profiling platforms, (3) building transparent AI models that clinicians can trust and (4) aligning with regulatory expectations for multi-OMIC companion diagnostics (CDx) and precision medicine (PMCDx). Achieving these goals will require close collaboration across rheumatology, oncology, multi-OMICs scientists, bioinformatics and regulatory science but the payoff is substantial: a new generation of immunological tools that can predict, prevent and precisely treat autoimmune disorders and treatment-related adverse effects.

The next phase of the field should focus less on discovering single universal biomarkers and more on building interoperable frameworks that combine systemic autoreactivity, tissue-specific pathology, and longitudinal clinical data. Progress will depend on standardized biospecimen pipelines, analytically validated reactome platforms, transparent multimodal models, and prospective evaluation in well-phenotyped cohorts. If successful, this approach could support a new generation of tools for risk prediction, endotyping, monitoring, and biomarker-guided intervention across both autoimmune disease and irAEs.

## Figures and Tables

**Table 1:** Simplified precision health biomarker framework across rheumatoid arthritis (RA) and immune checkpoint inhibitor-induced inflammatory arthritis (ICI-IA). The table summarizes major biomarker layers across the clinical trajectory, from baseline risk and early serology to high-dimensional autoantibody reactomes, tissue pathology, multimodal integration, and implementation requirements. Examples are shown for RA as a prototype autoimmune pathology and for ICI-IA as an induced immune toxicity model. It also highlights how multimodal integration and AI approaches can fuse tissue, blood, imaging, and clinical metadata to derive actionable outputs (risk scores, endotypes, and mechanistic inference) including CDx. Key references correspond to the manuscript reference list.

Clinical Stage/Use	Biomarker Layer	Sample/Source	What It Adds	RA Example/Use	ICI-IA/irAE Example/Use	Current Limitations/Evidence Level	Key References
Baseline risk	Genetic and clinical predisposition	Blood/saliva plus metadata	Host susceptibility and baseline context	HLA and exposure history refine risk context in RA	Baseline covariates may support irAE risk models	Moderate RA evidence; emerging irAE evidence; limited stand-alone predictive value.	[34,50,51]
Early immune dysregulation	Routine serology	Serum/plasma	Coarse evidence of loss of tolerance	RF and ACPA identify major RA subsets	Conventional autoantibodies have limited and inconsistent predictive value for irAEs	Established in RA; inconsistent and low–moderate predictive value in irAEs.	[12,13,29,30,31,52]
System-level immune profiling	Autoantibody reactome	Serum/plasma	High-dimensional autoreactivity landscape reflecting immune history	Broad panels may improve risk stratification and endotyping beyond RF/ACPA	Baseline or early on-treatment reactomes may predict toxicity and possibly treatment outcomes	Discovery-stage; needs platform standardization, thresholds, and external validation.	[18,43,53,54,55,56]
RA-focused mechanistic refinement	PTM-directed autoantibodies	Serum/plasma	Defines RA-relevant reactivity to modified self-antigens	ACPAs and other AMPAs help refine RA specificity and phenotype	Helps test whether ICI-IA is RA-like or biologically distinct	Strong RA rationale; exploratory in ICI-IA; PTM assays require harmonization.	[29,30,31,57,58]
Local pathology	Synovial histology and cellular composition	Synovial biopsy	Tissue organization and dominant immune–stromal programs	Supports tissue endotypes and links pathology to treatment response	May help distinguish transient from persistent ICI-IA	High mechanistic value; limited by cost	[16,36,42,59,60,61,62,63]
Tissue mechanism	Spatial and single-cell omics	Synovial tissue	Cell neighborhoods, active pathways, and pathogenic niches	Defines synovial microenvironments and tissue-resolved endotypes	May show how checkpoint blockade rewires joint pathology	Tissue access, small cohorts, and standardization.	[42,59,62,64,65,66,67,68]
Disease activity and monitoring	Circulating proteomics, EVs and cytokines	Serum/plasma	Dynamic systemic inflammatory state	Complements clinical scoring and monitoring	Commonly studied in irAE biomarker work, but strongest when integrated with other layers	Longitudinally useful but dynamic, non-specific, and treatment-sensitive.	[11,12,13,20,39,40,41]
Integrated prediction	Multimodal AI/multi-OMICs integration	Multi-source	Links tissue, blood, imaging, and clinical data into endotypes and risk scores	Supports flare prediction, endotype discovery, and treatment stratification	Supports prediction of irAEs, chronicity, and outcome trade-offs	Promising but exploratory; needs interpretability, calibration, and prospective validation.	[41,47,48,49,51,69,70,71,72,73,74]
Clinical implementation	Standardization, validation, and governance	Workflow/infrastructure	Enables real-world deployment and trust	Requires biopsy, assay, and imaging harmonization	Requires harmonized toxicity definitions, validation, and regulatory readiness	Requires validated workflows, governance, cost-effectiveness, and regulatory readiness.	[45,48,49,75,76,77,78,79,80]

## Data Availability

No new data were created or analyzed in this study.

## Abbreviations

The following abbreviations are used in this manuscript:

Abbreviation	Definition
AAbs	autoantibodies
AAgAtlas	Autoantigen Atlas
AAgs	autoantigens
AAPA	anti-acetylated protein antibodies
AAV	ANCA-associated vasculitis
ACPA	anti-citrullinated protein antibody
AI	artificial intelligence
AMPA	anti-modified protein antibody
ANA	antinuclear antibody
anti-CarP	anti-carbamylated protein antibodies
anti-CCP	anti-cyclic citrullinated peptide antibody
anti-dsDNA	anti-double-stranded DNA antibody
anti-MAA	anti-malondialdehyde-acetaldehyde adduct antibodies
anti-RNAP3	anti-RNA polymerase III antibody
CCP	cyclic citrullinated peptide
cDMARD	conventional disease-modifying anti-rheumatic drug
CDx	companion diagnostic(s)
CNN	convolutional neural network
COMP	cartilage oligomeric matrix protein
CRP	C-reactive protein
CT	computed tomography
CTGF	connective tissue growth factor
CTLA-4	cytotoxic T-lymphocyte-associated protein 4
DMARD	disease-modifying anti-rheumatic drug
ECM	extracellular matrix
EHR	electronic health record
ESR	erythrocyte sedimentation rate
EV	extracellular vesicle
FGFR	fibroblast growth factor receptor
GNN	graph neural network
HLA-B27	human leukocyte antigen B27
HLA-DRB1	human leukocyte antigen DR beta 1
IBD	inflammatory bowel disease
ICI	immune checkpoint inhibitor
ICI-IA	immune checkpoint inhibitor-induced inflammatory arthritis
IL-18	interleukin-18
IL-6	interleukin-6
IMC	imaging mass cytometry
irAE	immune-related adverse event
IVD	in vitro diagnostic(s)
LC-MS	liquid chromatography-mass spectrometry
LLM	large language model
MBDA	multi-biomarker disease activity
MIPSA	Molecular Indexing of Proteins by Self-Assembly
miRNA	microRNA
ML	machine learning
MMP-3	matrix metalloproteinase-3
MPO-ANCA	myeloperoxidase anti-neutrophil cytoplasmic antibody
MRI	magnetic resonance imaging
NGS	next-generation sequencing
PD-1	programmed cell death protein 1
PD-L1	programmed death-ligand 1
PhIP-seq	phage immunoprecipitation sequencing
PMCDx	precision medicine companion diagnostics
PMR	polymyalgia rheumatica
PR3-ANCA	proteinase 3 anti-neutrophil cytoplasmic antibody
PsA	psoriatic arthritis
PTM	post-translational modification
RA	rheumatoid arthritis
REAP	rapid extracellular antigen profiling
RF	rheumatoid factor
scRNA-seq	single-cell RNA sequencing
SLE	systemic lupus erythematosus
SLO	secondary lymphoid organ
SP	spatial proteomics
SSc	systemic sclerosis
ST	spatial transcriptomics
TGF-beta	transforming growth factor beta
TLS	tertiary lymphoid structure
TNF	tumor necrosis factor
TNF-alpha	tumor necrosis factor alpha
UGSB	ultrasound-guided synovial biopsy
VEGF	vascular endothelial growth factor
ViT	Vision Transformer
